# Second malignant tumors and non-tumor causes of death for patients with localized and regional kidney cancer after diagnosis

**DOI:** 10.1186/s40001-023-01176-6

**Published:** 2023-06-30

**Authors:** Luchen Yang, Xiaotian Wu, Jing Zhou, Pan Song, Zhenghuan Liu, Junhao Chen, Qiang Dong

**Affiliations:** 1 Department of Urology, Institution of Urology, West China Hospital of Sichuan University, No. 37, Guoxue Lane, Wuhou District, Sichuan Province 610041 Chengdu, China; 2 The First Clinical Medical College of Lanzhou University, Lanzhou, 730000 Gansu Province China

**Keywords:** Kidney cancer, SMT, Non-tumor causes of death, Localized, Regional, SEER

## Abstract

**Background:**

To evaluate the second malignant tumors (SMTs) and non-tumor causes of death among patients diagnosed with localized and regional kidney cancer.

**Methods:**

Patients diagnosed with kidney cancer between 2000 and 2017 in the Surveillance, Epidemiology, and End Results (SEER) program database were identified. All causes of death for patients during the follow-up and standardized mortality ratio (SMR) were analyzed.

**Result:**

113,734 patients with localized kidney cancer with 30,390 cases of death were analyzed. 60.4% of the death cases were due to non-tumor caused and 23.6% were second malignant tumors (SMTs). Main SMTs included cancers of lung and bronchus [*n* = 1,283, SMR: 1.00 (0.95–1.06)] and pancreas [*n* = 393, SMR: 1.27 (1.15–1.41)]. Causes of death for non-tumor mainly included heart diseases [*n* = 6,161, SMR: 1.25 (1.21–1.28)] and chronic obstructive pulmonary disease (COPD) [*n* = 1,185, SMR: 0.99 (0.94–1.05)]. 14,437 of 29,602 patients with regional kidney cancer died. 14.6% of all deaths were due to SMTs and 23.6% due to non-tumor causes. Main SMTs contained bladder cancer [*n* = 371, SMR: 10.90 (9.81–12.06)] and lung and bronchus cancer [*n* = 346, SMR: 1.21 (1.08–1.34)]. The main non-tumor death was heart disease [*n* = 1,424, SMR: 1.26 (1.2–1.33)]. When stratified by pathological types, patients with clear cell renal cell carcinoma (RCC) did not have increased mortality risks of bladder cancer and lung cancer but patients with non-clear cell RCC did.

**Conclusion:**

SMTs and non-tumor diseases including lung and bronchus cancer, bladder cancer, pancreas cancer, diseases of heart, COPD, and cerebrovascular diseases are the leading causes of death besides kidney cancer and should be paid more attention during patients’ survival period.

**Supplementary Information:**

The online version contains supplementary material available at 10.1186/s40001-023-01176-6.

## Introduction

Kidney cancer, the 13th most common malignant tumor in the world, accounts for 2.4% of all malignant tumor cases [1]. It is estimated that there are about 79,000 new cases of kidney cancer and 13,920 deaths globally in 2022 [2]. The incidence of kidney cancer has been increasing globally at an annual rate of approximately 2–3% since the 1970s [3]. But its mortality rate has remained stable since the 1990s [4]. Most newly diagnosed cases are at the localized stage and are potentially curative by surgical resection with an excellent survival outcome [5, 6]. For the treatment of localized renal cell cancer (RCC), partial nephrectomy for small tumors and radical nephrectomy for large tumors remains gold standard treatments. Many factors like patients’ age, tumor size is, and ischemia time that could affect postoperative renal function, quality of life and even the survival outcomes [7, 8]. The survival of patients with regional or distant metastasis kidney cancer was also greatly improved with the continuous optimization of treatment regimens. The 5-year overall survival for kidney cancer patients has also been improved from 57% in the late 1980s to > 70% in recent years [9].

The etiologies of kidney cancer include genetic factors, environmental and occupational exposure, medical history, lifestyle, and other factors [1]. Some of them may also contribute to the development of second malignant tumors (SMTs). In addition, the incidence of kidney cancer increases with age, and is prone to occur in the elderly [4]. Elderly patients are more likely to have comorbidities that increase their risk of dying from some underlying diseases like hypertension, coronary heart disease, etc. It is meaningful to explore all causes of death and related mortality risks for kidney cancer patients. To the best of our knowledge, no study in the current literature has evaluated the causes of death from SMTs and non-neoplastic diseases among kidney cancer patients. In this study, we aimed to evaluate all causes of death (kidney cancer, SMTs, and non-tumor diseases) for patients with localized and regional kidney cancer, and to calculate the mortality risks of each cause compared with the general population.

## Materials and methods

### Data sources

The data of the retrospective cohort study were obtained from the Surveillance, Epidemiology, and End Results (SEER) program database, which provides information on population-based cancer incidence and survival data from approximately 28% of the US population. The mortality data of the general population during 2000–2017 were drawn from the National Center for Health Statistics. The kidney cancer incidence and mortality of the Americans were from the Global Cancer Observatory (GCO) which is based on the GLOBOCAN estimates of incidence, mortality and prevalence in 185 countries or territories for 36 cancer types.

### Study population and study variables

Patients who were diagnosed with kidney cancer according to ICD-O-3/WHO 2008 definition between 2000 and 2017 were included. Inclusion criteria: ① kidney cancer was the only one or the first of multiple primary malignant tumors; ② all patients had definite staging and pathological information; ③ the follow-up time, living status (alive or dead), and the detailed causes of death were clear. Exclusion criteria: ① patients with synchronous multiple tumors with the cutoff of 2 months in accordance with SEER criteria [10]; ② the follow-up time was 0; ③ patients died within 2 months after the diagnosis of kidney cancer.

For included patients, the following demographic and clinical variables were collected: age at diagnosis (15–44 years, 45–54 years, 55–64 years, 65–74 years, 75 + years), year of diagnosis (2000–2006, 2007–2012, and 2013–2017), race (white, black, and others), grade (well differentiated, moderate differentiation, poor differentiation, and undifferentiated), main pathological type, surgical therapy (partial nephrectomy, radical nephrectomy, local tumor destruction), chemotherapy (yes, no/unknown). The last follow-up time was December 31, 2018.

### Outcome assessments

Our study evaluated all causes of death that were identified from death codes in the SEER. The mortality risks were assessed by the standardized mortality ratio (SMR), which was calculated by comparing the observed number of deaths to the expected one. The expected number of deaths was based on the total person-year of included patients and the mortality rate of all causes of death among the general population.

All causes of death and related SMR were regarded as the primary outcomes of our study. The incidence and mortality from 2000 to 2017, estimated incidence and mortality up to 2040 and rates in different age groups were defined as the second outcomes.

### Statistical analyses

Patients were segregated into two groups according to their stage information (localized or regional). With the incidence-SEER research data, 18 registries database and standardized incidence ratios tables function in the SEER*Stat 8.40 software, we analyzed all causes of death (114 items) and calculated SMRs with its 95% confidence intervals (95%CI) with the exact method of statistics. The various mortality rates of the US general population were extracted from the National Center for Health Statistics that was available through the SEER program. We also estimated their SMRs and 95%CI for patients with different pathological types (clear cell RCC or non-clear RCC) and different survival times after diagnosis (< 5 years, 5–10 years, > 10 years). The mortality rates were calculated by the death of different causes and total number of patients each year during the follow-ups. *P* < 0.05 was considered to be statistically significant.

## Results

A total of 143,336 patients were identified, and 44,827 of them died during the follow-up. The majority of patients (*n* = 113,734) had localized disease with a total mortality rate of 27% (*n* = 30,390). 29,602 patients were in the regional stage whose mortality rate was 49% (*n* = 14,437). The baseline characteristics of all patients and all deaths in localized and regional stages are presented in Table 1.

**Table 1:** Baseline characteristics of included patients

Variables	Localized			Regional		
	Patients, *n*	Deaths, *n*	SMR (95% CI)	Patients, *n*	Deaths, *n*	SMR (95% CI)
Total	11,3734	30,390	1.59^#^ (1.57–1.6)	29,602	14,437	3.36^#^ (3.31–3.42)
Age						
15–44 years	13,137	1,041	3.24^#^ (3.04–3.44)	1,843	607	17.15^#^ (15.82–18.57)
45–54 years	22,692	3,265	2.44^#^ (2.35–2.52)	4,646	1,739	8.07^#^ (7.7–8.46)
55–64 years	31,595	6,713	1.97^#^ (1.93–2.02)	7,958	3,361	5.04^#^ (4.87–5.21)
65–74 years	27,606	8,969	1.51^#^ (1.48–1.54)	8,112	4,222	3.17^#^ (3.07–3.26)
75 + years	17,619	10,361	1.27^#^ (1.25–1.3)	6,246	4,451	2.18^#^ (2.12–2.25)
Sex						
Male	69,116	18,873	1.56^#^ (1.54–1.59)	19,456	9,371	3.22^#^ (3.15–3.28)
Female	44,618	11,517	1.62^#^ (1.59–1.65)	10,146	5,066	3.67^#^ (3.57–3.77)
Year of diagnosis						
2000–2007	39,932	18,042	1.58^#^ (1.55–1.6)	11,362	7,905	3.16^#^ (3.09–3.23)
2008–2012	34,484	8,521	1.57^#^ (1.54–1.61)	8,553	4,133	3.43^#^ (3.33–3.54)
2013–2017	39,318	3,827	1.67^#^ (1.61–1.72)	9,687	2,399	4.10^#^ (3.94–4.27)
Race						
White	92,846	24,884	1.52^#^ (1.51–1.54)	25,262	12,322	3.20^#^ (3.15–3.26)
Black	14,168	4,073	1.86^#^ (1.81–1.92)	2,400	1,274	4.61^#^ (4.36–4.87)
American Indian-Alaska Native	891	247	4.19^#^ (3.69–4.75)	237	97	6.43^#^ (5.21–7.84)
Asian or Pacific Islander	5,829	1,186	2.04^#^ (1.93–2.16)	1,703	744	4.84^#^ (4.5–5.21)
Grade						
Well differentiated	11,586	2,302	1.39^#^ (1.33–1.45)	1,102	465	2.11^#^ (1.92–2.31)
Moderately differentiated	42,645	7,706	1.33^#^ (1.3–1.36)	7,879	3,059	2.15^#^ (2.07–2.22)
Poorly differentiated	18,250	4,089	1.62^#^ (1.57–1.67)	9,432	4,718	3.60^#^ (3.5–3.71)
Undifferentiated	3,213	1,147	2.48^#^ (2.34–2.62)	5,275	3,164	5.68^#^ (5.48–5.88)
Pathological type						
Papillary	13,202	2,996	1.38^#^ (1.33–1.43)	1,691	758	2.91^#^ (2.71–3.13)
Chromophores	6,352	908	0.92^#^ (0.86–0.98)	1,005	254	1.67^#^ (1.47–1.88)
Clear cell	58,472	12,549	1.47^#^ (1.45–1.5)	14,201	5,660	2.85^#^ (2.78–2.93)
Surgical treatment						
Partial nephrectomy	33,344	3,535	1.02 (0.99–1.06)	2,339	569	1.60^#^ (1.47–1.74)
Radical nephrectomy	48,672	11,929	1.61^#^ (1.58–1.64)	24,595	11,902	3.27^#^ (3.21–3.33)
Local tumor destruction	5,983	1,527	1.35^#^ (1.29–1.42)	128	78	3.44^#^ (2.72–4.29)
Surgery, NOS	933	260	1.63^#^ (1.44–1.84)	410	215	3.22^#^ (2.81–3.69)
No	7,084	3,933	3.11^#^ (3.01–3.21)	1,551	1,328	9.84^#^ (9.31–10.38)
Chemotherapy						
Yes	1,732	453	5.29^#^ (4.81–5.8)	3,562	1,841	8.96^#^ (8.55–9.38)
No/unknown	112,002	29,937	1.57^#^ (1.55–1.59)	26,040	12,596	3.08^#^ (3.03–3.14)

*SMR* standardized mortality ratio, *CI* confidence interval, *NOS* not otherwise specified

^#^Statistical significance with P < 0.05

**Fig. 1:**
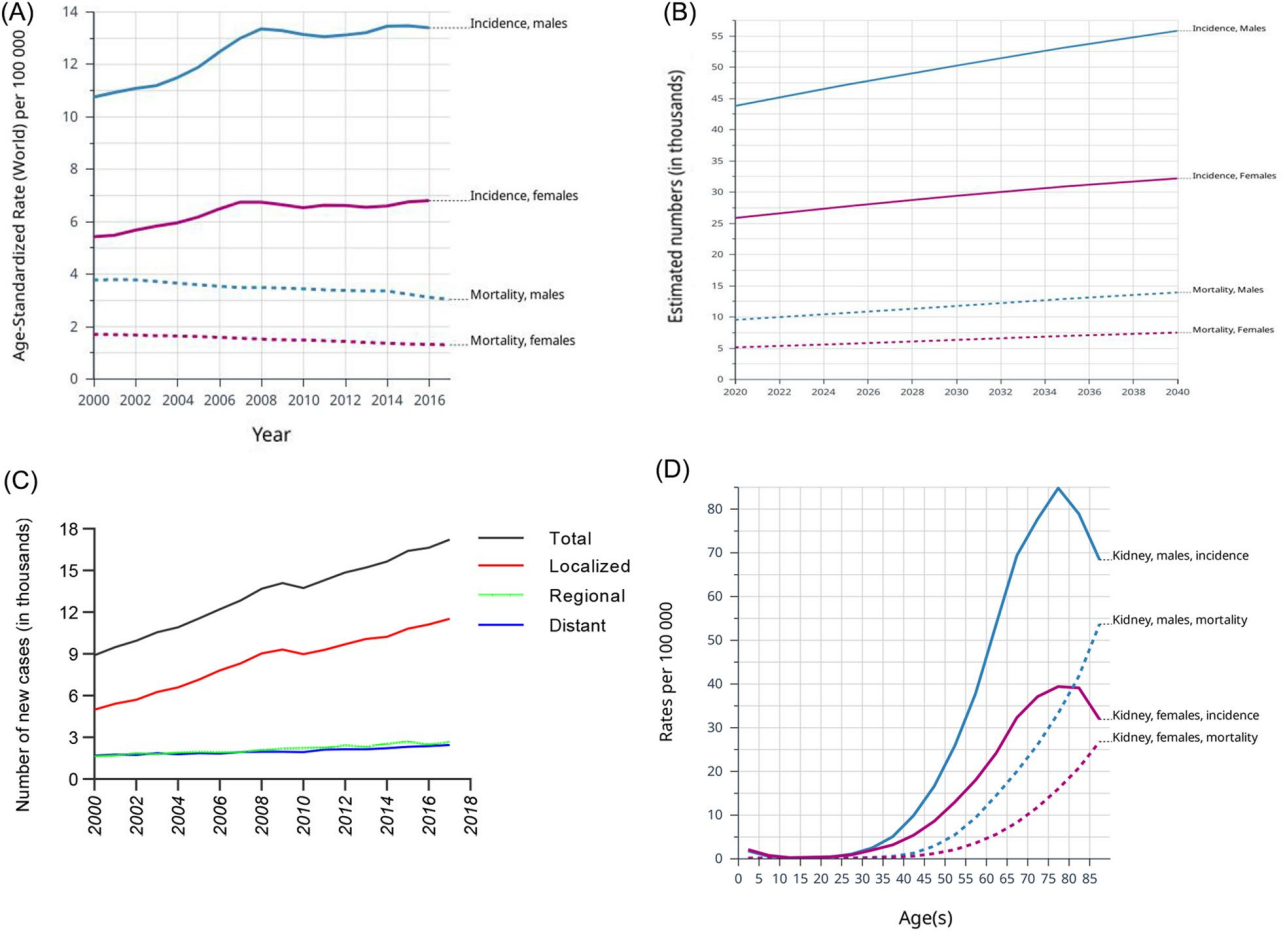
The incidence and mortality of kidney cancer in the United States. **A** Kidney cancer incidence and mortality, 2000–2017. **B** Estimated incidence and mortality of kidney cancer patients in 2020–2040. **C** The number of new kidney cancer cases diagnosis in different stages from 2000 to 2017. **D** Incidence and mortality of kidney cancer patients in different age groups

The incidence and mortality among patients with kidney cancer from 2000 to 2017 as well as the estimated number of incidence and mortality up to 2040 are presented in Fig. 1A and B. The newly diagnosed cases in different stages showed that the increasing incidence of total kidney cancer was mainly due to the increase of localized diseases (Fig. 1C). The mortality remained stable from 2000 to 2017 as well as in the estimated future. The incidence and mortality of patients with different ages between 2000 and 2017 are presented in Fig. 1D.

### Causes of death for patients with localized disease

**Fig. 2:**
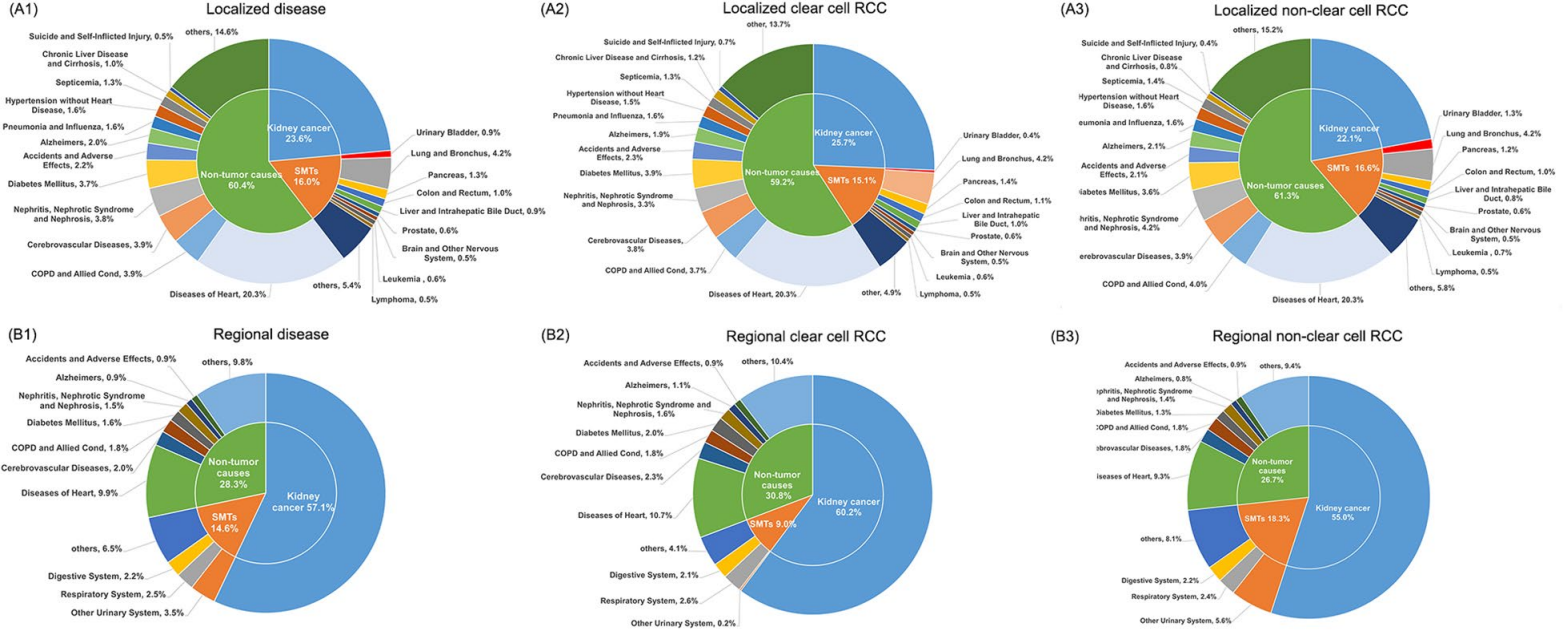
The percentages of main causes of death in kidney cancer patients. **A1** Patients with localized kidney cancer. **A2** Patients with localized clear cell RCC. **A3** Patients with localized non-clear cell RCC. **B1** Patients with regional kidney cancer. **B2** Patients with regional clear cell RCC. **B3** Patients with regional non-clear cell RCC

**Fig. 3:**
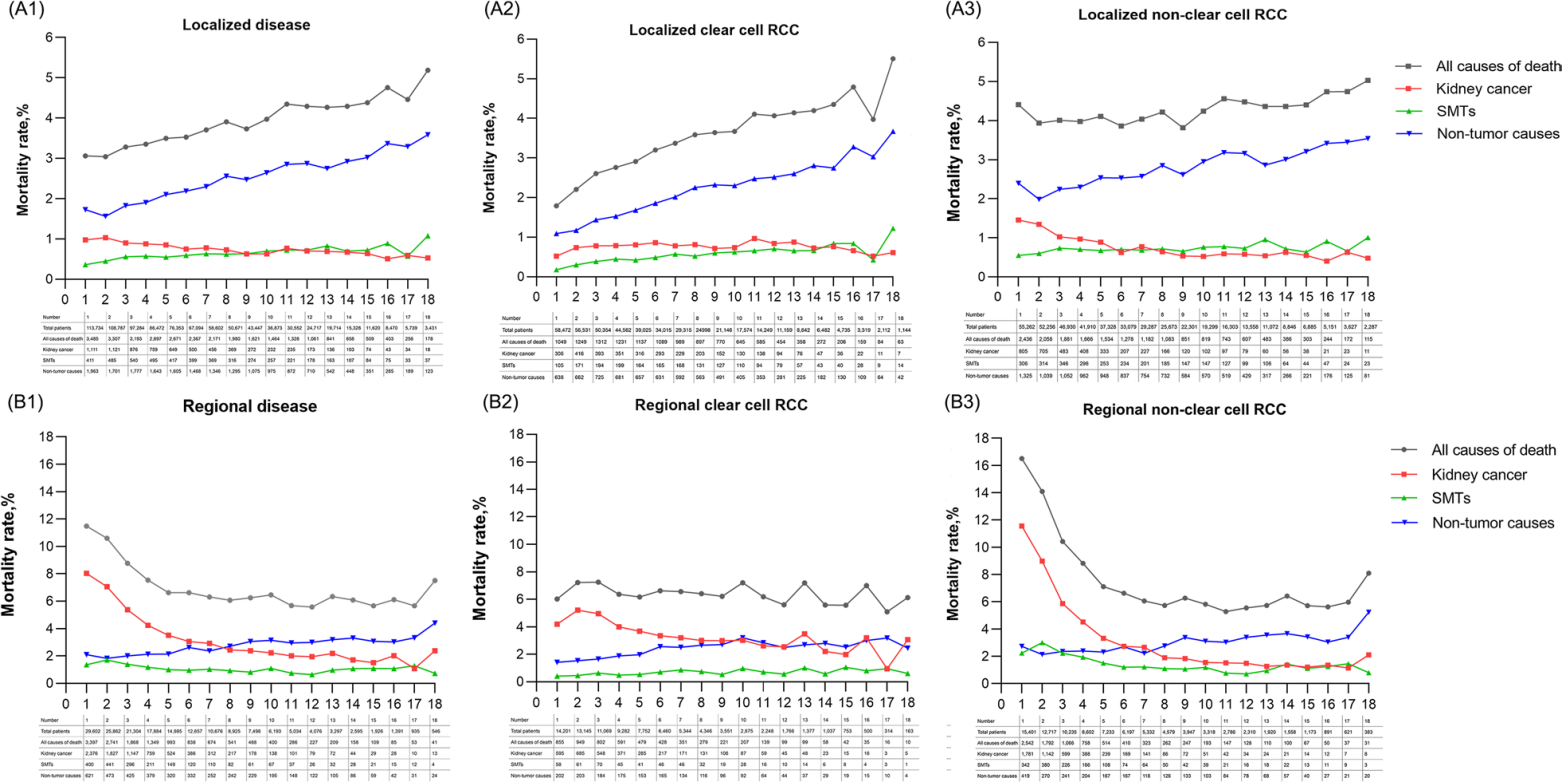
The mortality rate of main causes (including all causes of death, kidney cancer, SMTs and non-tumor causes) of death among patients with localized and regional kidney cancer with the year after diagnosis. **A1** Mortality rate of main causes among patients with localized disease. **A2** Mortality rate of main causes among patients with localized clear cell RCC. **A3** Mortality rate of main causes among patients with localized non-clear cell RCC. **B1** Mortality rate of main causes among patients with regional disease. **B2** Mortality rate of main causes among patients with regional clear cell RCC. **B3** Mortality rate of main causes among patients with regional non-clear cell RCC

A total of 113,734 patients with localized kidney cancer were identified, and 30,390 of them died. 15,553(51.2%) of all deaths occurred within 5 years, 9,603(31.6%) and 5,234(17.2%) deaths were observed in the periods of 5–10 years and > 10 years after diagnosis. Kidney cancer deaths only accounted for 23.6% of all deaths, SMTs accounted for 16.0% and the remaining 60.4% were due to non-tumor causes. Main SMTs included cancer from the lung and bronchus [*n* = 1,283, SMR: 1.00 (0.95–1.06)], pancreas [*n* = 393, SMR: 1.27 (1.15–1.41)], colon and rectum [*n* = 315, SMR: 0.78 (0.7–0.87)], liver and intrahepatic bile duct [*n* = 275, SMR: 1.50 (1.33–1.69)], and urinary bladder [*n* = 277, SMR: 1.95 (1.73–2.2)]. The main causes of non-tumor deaths included heart diseases [*n* = 6,161, SMR: 1.25 (1.21–1.28)], chronic obstructive pulmonary disease (COPD) and allied cond [*n* = 1,185, SMR: 0.99 (0.94–1.05)], cerebrovascular diseases [*n* = 1,183, SMR: 1.12 (1.06–1.18)], nephritis, nephrotic syndrome and nephrosis [*n* = 1,166, SMR: 3.01 (2.84–3.18)], and diabetes mellitus [*n* = 1,137, SMR: 1.91 (1.8–2.02)]. When stratified by pathological types, RCC patients had significantly lower risk of bladder cancer than these with non-clear RCC, as well as lower lung and bronchus cancer and COPD and allied cond mortality risks. For both patients with clear cell and non-clear cell kidney cancer, there was a significant increase in the SMR of cancer from pancreas, liver and intrahepatic bile duct, as well as brain and other nervous systems. The mortality risk of prostate cancer, colon and rectum cancer decreased significantly. All these results are presented in Table 2, Fig. 2A and Additional file 1. The mortality rates of all causes of death, kidney cancer, SMTs and non-tumor causes are presented in Fig. 3A. There were no significant differences in mortality rate from SMTs and non-neoplastic causes between the clear cell and non-clear cell RCC groups.

**Table 2:** Main causes of death for patients with localized kidney cancer

Causes of death	Total			Clear cell RCC			Non-clear cell RCC		
	Observed, *n*	Expected, *n*	SMR (95% CI)	Observed, *n*	Expected, *n*	SMR (95% CI)	Observed, *n*	Expected, *n*	SMR (95% CI)
All causes of death	30,390	19,151.73	1.59^#^ (1.57–1.6)	12,549	8,512.19	1.47^#^ (1.45–1.5)	17,841	10,639.55	1.68^#^ (1.65–1.7)
All malignant cancers	12,022	4,575.66	2.63^#^ (2.58–2.67)	5,118	2,095.27	2.44^#^ (2.38–2.51)	6,904	2,480.39	2.78^#^ (2.72–2.85)
Urinary system	7,500	260.56	28.78^#^ (28.14–29.44)	3,267	117.66	27.77^#^ (26.82–28.74)	4,233	142.9	29.62^#^ (28.74–30.53)
Kidney and renal pelvis	7,161	111.5	64.22^#^ (62.74–65.73)	3,220	51.54	62.48^#^ (60.34–64.67)	3,941	59.96	65.73^#^ (63.69–67.81)
Urinary bladder	277	141.91	1.95^#^ (1.73–2.2)	44	62.87	0.70^#^ (0.51–0.94)	233	79.03	2.95^#^ (2.58–3.35)
Respiratory system	1,315	1,318.67	1 (0.94–1.05)	538	606.11	0.89^#^ (0.81–0.97)	777	712.56	1.09^#^ (1.02–1.17)
Lung and bronchus	1,283	1,277.13	1 (0.95–1.06)	529	587.53	0.90^#^ (0.83–0.98)	754	689.6	1.09^#^ (1.02–1.17)
Digestive system	1,276	1,176.37	1.08^#^ (1.03–1.15)	566	540.52	1.05 (0.96–1.14)	710	635.85	1.12^#^ (1.04–1.2)
Pancreas	393	308.78	1.27^#^ (1.15–1.41)	174	143.53	1.21^#^ (1.04–1.41)	219	165.25	1.33^#^ (1.16–1.51)
Colon and rectum	315	403.3	0.78^#^ (0.7–0.87)	140	181.26	0.77^#^ (0.65–0.91)	175	222.04	0.79^#^ (0.68–0.91)
Liver and intrahepatic bile duct	275	183.41	1.50^#^ (1.33–1.69)	126	86.78	1.45^#^ (1.21–1.73)	149	96.63	1.54^#^ (1.3–1.81)
Male genital system	183	324.19	0.56^#^ (0.49–0.65)	72	132.98	0.54^#^ (0.42–0.68)	111	191.22	0.58^#^ (0.48–0.7)
Prostate	178	319.62	0.56^#^(0.48–0.65)	71	130.87	0.54^#^ (0.42–0.68)	107	188.74	0.57^#^ (0.46–0.69)
Brain and other nervous system	159	100.52	1.58^#^(1.35–1.85)	68	49.1	1.38^#^ (1.08–1.76)	91	51.42	1.77^#^ (1.42–2.17)
Leukemia	190	182.42	1.04 (0.9–1.2)	72	82.78	0.87 (0.68–1.1)	118	99.64	1.18 (0.98–1.42)
Lymphoma	148	173.82	0.85 (0.72–1)	62	79.52	0.78^#^ (0.6–1)	86	94.3	0.91 (0.73–1.13)
Non-tumor causes									
Diseases of heart	6,161	4,945.61	1.25^#^ (1.21–1.28)	2,547	2,140.78	1.19^#^ (1.14–1.24)	3,614	2,804.84	1.29^#^ (1.25–1.33)
COPD and allied cond	1,185	1,194.07	0.99 (0.94–1.05)	470	543.39	0.86^#^ (0.79–0.95)	715	650.67	1.10^#^ (1.02–1.18)
Cerebrovascular diseases	1,183	1,058.31	1.12^#^ (1.06–1.18)	480	455.48	1.05 (0.96–1.15)	703	602.83	1.17^#^ (1.08–1.26)
Nephritis, nephrotic syndrome and nephrosis	1,166	387.89	3.01^#^ (2.84–3.18)	409	165.73	2.47^#^ (2.23–2.72)	757	222.16	3.41^#^ (3.17–3.66)
Diabetes mellitus	1,137	595.36	1.91^#^ (1.8–2.02)	488	265.59	1.84^#^ (1.68–2.01)	649	329.77	1.97^#^ (1.82–2.13)
Accidents and adverse effects	668	602.41	1.11^#^ (1.03–1.2)	293	282.38	1.04 (0.92–1.16)	375	320.03	1.17^#^ (1.06–1.3)
Alzheimer’s	611	701.64	0.87^#^ (0.8–0.94)	242	300.21	0.81^#^ (0.71–0.91)	369	401.43	0.92 (0.83–1.02)
Pneumonia and influenza	489	429.8	1.14^#^ (1.04–1.24)	197	184.8	1.07 (0.92–1.23)	292	245	1.19^#^ (1.06–1.34)
Hypertension without heart disease	482	223.68	2.15^#^ (1.97–2.36)	192	96.06	2.00^#^ (1.73–2.3)	290	127.62	2.27^#^ (2.02–2.55)
Septicemia	406	285.54	1.42^#^ (1.29–1.57)	164	125.48	1.31^#^ (1.11–1.52)	242	160.05	1.51^#^ (1.33–1.71)
Chronic liver disease and cirrhosis	293	220.44	1.33^#^ (1.18–1.49)	149	110.01	1.35^#^ (1.15–1.59)	144	110.44	1.30^#^ (1.1–1.54)
Suicide and self-inflicted injury	151	153.78	0.98 (0.83–1.15)	83	78.18	1.06 (0.85–1.32)	68	75.61	0.9 (0.7–1.14)

*SMR* standardized mortality ratio, *CI* confidence interval, *COPD* chronic obstructive pulmonary disease

^#^Statistical significance with P < 0.05

### Causes of death for patients with regional disease

Among the 29,602 patients with regional disease, 14,437 of them died during the follow-up. 10,348(71.7%) and 4089(28.3%) deaths occurred during the periods of < 5 years and > 5 years, respectively. 8,240 (57.1%) of all deaths were due to kidney cancer. SMTs and non-tumor causes accounted for 14.6% and 28.3% of all deaths, individually. Main SMTs included urinary bladder [*n* = 371, SMR: 10.90 (9.81–12.06)], lung and bronchus [*n* = 346, SMR: 1.21 (1.08–1.34)], and pancreas [*n* = 106, SMR: 1.57 (1.29–1.9)]. The main non-tumor causes included diseases of heart [*n* = 1,424, SMR: 1.26 (1.2–1.33)], cerebrovascular diseases [*n* = 289, SMR: 1.21 (1.08–1.36)], and diabetes mellitus [*n* = 227, SMR: 1.76 (1.54–2.0)]. The SMRs of deaths due to accidents, adverse effects, suicide and self-inflicted injury were not significantly improved. Patients with clear cell RCC did not exhibit an increased mortality risk of bladder cancer, lung and bronchus cancer and pancreas cancer, but patients with non-clear cell RCC did. Both groups had decreased mortality risk of prostate cancer. These results are presented in Table 3, Fig. 2B and Additional file 1. Mortality rates from SMT and non-tumor causes were similar in both groups and stabilized over time (Fig. 3B).

**Table 3:** Main causes of deaths for patients with regional kidney cancer

Causes of deaths	Total			Clear cell RCC			Non-clear cell RCC		
	Observed, *n*	Expected, *n*	SMR (95% CI)	Observed, *n*	Expected, *n*	SMR (95% CI)	Observed, *n*	Expected, *n*	SMR (95% CI)
All causes of death	14,437	4,292.43	3.36^#^ (3.31–3.42)	5,660	1,983.35	2.85^#^ (2.78–2.93)	8,777	2,309.08	3.80^#^ (3.72–3.88)
All malignant cancers	10,352	1,016.84	10.18^#^ (9.99–10.38)	3,918	487.27	8.04^#^ (7.79–8.3)	6,434	529.57	12.15^#^ (11.85–12.45)
Urinary system	8,746	61.07	143.22^#^ (140.24–146.26)	3,423	29.15	117.44^#^ (113.54–121.44)	5,323	31.92	166.77^#^ (162.32–171.31)
Kidney and renal pelvis	8,240	25.34	325.20^#^ (318.21–332.3)	3,410	12.36	275.95^#^ (266.77–285.37)	4,830	12.98	372.08^#^ (361.66–382.72)
Urinary bladder	371	34.05	10.90^#^ (9.81–12.06)	10	15.99	0.63 (0.3–1.15)	361	18.06	19.99^#^ (17.98–22.16)
Ureter	57	0.8	71.17^#^ (53.91–92.21)	0	0.38	0 (0–9.7)	57	0.42	135.53^#^ (102.65–175.59)
Respiratory system	354	295.99	1.20^#^ (1.07–1.33)	145	142.81	1.02 (0.86–1.19)	209	153.19	1.36^#^ (1.19–1.56)
Lung and bronchus	346	286.72	1.21^#^ (1.08–1.34)	140	138.32	1.01 (0.85–1.19)	206	148.41	1.39^#^ (1.2–1.59)
Digestive System	311	259.38	1.20^#^ (1.07–1.34)	119	125.56	0.95 (0.79–1.13)	192	133.83	1.43^#^ (1.24–1.65)
Pancreas	106	67.4	1.57^#^ (1.29–1.9)	36	32.89	1.09 (0.77–1.52)	70	34.51	2.03^#^ (1.58–2.56)
Colon and rectum	87	89.3	0.97 (0.78–1.2)	35	41.94	0.83 (0.58–1.16)	52	47.36	1.1 (0.82–1.44)
Liver and intrahepatic bile duct	48	39.77	1.21 (0.89–1.6)	23	20.15	1.14 (0.72–1.71)	25	19.61	1.27 (0.82–1.88)
Male genital system	46	78.63	0.59^#^ (0.43–0.78)	18	35.37	0.51^#^ (0.3–0.8)	28	43.26	0.65^#^ (0.43–0.94)
Prostate	43	77.58	0.55^#^ (0.4–0.75)	17	34.85	0.49^#^ (0.28–0.78)	26	42.73	0.61^#^ (0.4–0.89)
Soft tissue including heart	50	6.27	7.98^#^ (5.92–10.51)	2	3.08	0.65 (0.08–2.35)	48	3.19	15.05^#^ (11.1–19.96)
Brain and other nervous system	44	22	2.00^#^ (1.45–2.68)	21	11.15	1.88^#^ (1.17–2.88)	23	10.86	2.12^#^ (1.34–3.18)
Leukemia	35	42.31	0.83 (0.58–1.15)	10	20.05	0.50^#^ (0.24–0.92)	25	22.25	1.12 (0.73–1.66)
Lymphoma	29	40.39	0.72 (0.48–1.03)	13	19.15	0.68 (0.36–1.16)	16	21.24	0.75 (0.43–1.22)
Non-tumor causes									
Diseases of heart	1,424	1,128.76	1.26^#^ (1.2–1.33)	607	509.76	1.19^#^ (1.1–1.29)	817	619	1.32^#^ (1.23–1.41)
Cerebrovascular diseases	289	238.34	1.21^#^ (1.08–1.36)	129	105.6	1.22^#^ (1.02–1.45)	160	132.73	1.21^#^ (1.03–1.41)
COPD and allied cond	266	274.97	0.97 (0.85–1.09)	104	129.15	0.81^#^ (0.66–0.98)	162	145.82	1.11 (0.95–1.3)
Diabetes mellitus	227	129.1	1.76^#^ (1.54–2)	112	60.84	1.84^#^ (1.52–2.22)	115	68.25	1.68^#^ (1.39–2.02)
Nephritis, nephrotic syndrome and nephrosis	210	85.61	2.45^#^ (2.13–2.81)	90	38.64	2.33^#^ (1.87–2.86)	120	46.97	2.56^#^ (2.12–3.06)
Pneumonia and influenza	138	100	1.38^#^ (1.16–1.63)	48	44.17	1.09 (0.8–1.44)	90	55.82	1.61^#^ (1.3–1.98)
Alzheimer’s	130	159.43	0.82^#^ (0.68–0.97)	60	69.31	0.87 (0.66–1.11)	70	90.13	0.78^#^ (0.61–0.98)
Accidents and adverse effects	130	128.41	1.01 (0.85–1.2)	53	61.93	0.86 (0.64–1.12)	77	66.49	1.16 (0.91–1.45)
Septicemia	108	61.91	1.74^#^ (1.43–2.11)	39	28.59	1.36 (0.97–1.86)	69	33.32	2.07^#^ (1.61–2.62)
Hypertension without heart disease	89	47.89	1.86^#^ (1.49–2.29)	37	21.55	1.72^#^ (1.21–2.37)	52	26.34	1.97^#^ (1.47–2.59)
Chronic liver disease and cirrhosis	65	45.89	1.42^#^ (1.09–1.81)	35	24.07	1.45^#^ (1.01–2.02)	30	21.82	1.37 (0.93–1.96)
Suicide and self-inflicted injury	33	32.71	1.01 (0.69–1.42)	15	17.1	0.88 (0.49–1.45)	18	15.62	1.15 (0.68–1.82)

*SMR* standardized mortality ratio, *CI* confidence interval, *COPD* chronic obstructive pulmonary disease

^#^Statistical significance with P < 0.05

## Discussion

In our study, we found that the increased incidence of kidney cancer from 2000 to 2017 was predominantly attributed to the growth of localized diseases. Because of the wide use of imaging technology including ultrasound, CT, MRI, etc., many early-stage kidney cancer were found by unintentional detection [11, 12]. A previous study reported that RCC incidence increased while renal pelvis cancer rate decreased over time in the US [13]. Our data showed that the incidence of kidney cancer increase with age and reached high levels after the age of 60 in both men and women. The incidence and mortality in females were obviously lower than that in males [12, 14]. It was reported that about half of all cases of kidney cancer were diagnosed among patients above 65 years old [1].

For the SMTs among kidney cancer patients, our results found that the most common SMTs were from lung and bronchus cancer, pancreas cancer, colon and rectum cancer, bladder cancer, prostate cancer, leukemia (mainly myeloid and monocytic leukemia), brain and other nervous system tumor and lymphoma (mainly non-Hodgkin’s lymphomas). A previous study based on 1,425 RCC cases with multiple primary cancers found that prostate, bladder, lung, breast, colon and rectal cancer, malignant melanomas and non-Hodgkin’s lymphomas were the most common SMTs [15]. An international collaborative study with a median of 11.6 years follow-up period found the standardized incidence ratios for solid tumors and leukemias after Wilms tumor were 5.1 and 5.0, respectively [16]. The most common SMTs were the digestive organs, breast, thyroid, bone and central nervous system. Another study with 447 patients undergoing nephrectomy for the primary renal tumor in Croatia found that the most common SMTs were prostate and colon carcinoma [17]. The reason these results differed from ours was that our study focused on the mortality of SMTs rather than the incidence. Although some SMTs such as pancreatic cancer and leukemia, were not common, their high mortality rates warrant sufficient attention.

Compared with the general population, our results showed that kidney cancer patients were associated with increased mortality risks in SMTs like pancreas cancer, liver and intrahepatic bile duct cancer, stomach cancer and nervous system tumors, and decreased mortality risk in prostate cancer and colon and rectum cancer. Some studies showed increased risks of lung, bladder, rectal, prostate, thyroid gland, adrenal gland and nervous system cancer and non-Hodgkin’s lymphoma in primary RCC patients [18–20]. A bidirectional association was found between thyroid cancer, breast cancer and kidney cancer, with a threefold and 1.5-fold increase in the prevalence of thyroid cancer in men and women with kidney cancer, respectively [21]. Our results firstly found that the increased mortality risk of bladder cancer among kidney cancer patients was due to the non-clear cell RCC patients. The patients with clear cell RCC patients did not have increased risks of bladder cancer when comparing with general population.

Among all causes of death, our results found that non-tumor causes accounted for 60.4% and 28.3% of localized and regional diseases. The most common causes included heart diseases, COPD and allied cond, cerebrovascular diseases, nephritis, nephrotic syndrome and nephrosis, and diabetes mellitus. In the current literature, there were few studies that evaluated the non-tumor causes of death among kidney cancer patients. An Australia study analyzed the non-cancer causes of death among all cancer patients [22]. Their results showed that nearly 50% of cancer patients were more likely to die of non-cancer causes and the major non-cancer causes included cardiovascular disease (coronary heart disease and stroke), respiratory diseases, diseases of the digestive system, injury and poisoning and endocrine, nutritional and metabolic diseases [22]. A study reported that cardiovascular disease death cases accounted for a more significant proportion than those who died of RCC (26.4% vs 22.5%) for RCC patients with localized stage [23]. Another study with the SEER data [3] showed that the most common non-cancer causes of death among all cancer patients was the heart disease, which accounted above 40% of all deaths [3]. In our study, the heart diseases accounted for 20.3% and 9.9% of all localized and regional kidney cancer deaths. Another study with Korean survivors of adult cancer showed that the leading specific non-tumor causes of death among all cancer patients were cerebrovascular disease, diabetes mellitus, ischemic heart disease, suicide, and chronic lower respiratory disease [24].

Our results showed that the mortality risks of heart diseases, cerebrovascular diseases, nephritis, nephrotic syndrome and nephrosis, diabetes mellitus, pneumonia and influenza, hypertension, septicemia, and chronic liver disease were significantly increased in all kidney cancer patients when comparing with general population. While the mortality risk of COPD was only increased in the non-clear cell RCC patients but not in clear cell RCC patients. All kidney patients were associated with lower mortality risk of Alzheimer. Increased mortality risk in some diseases might due to the impaired kidney function. The reduced kidney function was closely associated with the increased deaths from heart failure, valvular diseases, infectious and other causes [25]. It was reported that the mortality of sepsis had relation with the cancer. The mortality of cancer-related sepsis was 27.9% which was significantly higher than 19.5% of non-tumor-related sepsis [26]. There were some different results in previous studies. In Zaorsky et al. study [3], the SMR of above non-cancer causes were all significantly increased among cancer patients. The SMR of Alzheimer only decreased in the period of 2–11 months and 12–59 months after diagnosis. Besides, their SMR values were obviously higher than ours. However, in Shin et al. study [24], the risk for non-cancer death was significantly lower among long-term survivors (SMR, 0.78; 95% CI 0.76–0.80). The specific causes of death including diabetes mellitus, hypertensive diseases, heart diseases, and cerebrovascular disease, etc., were all decreased. The reason for the different mortality risk in different studies might due to the patient’s inclusion. Because there were so many causes of death, if there were not enough patients, the number of patients for each specific cause would be very limited. The results would be influenced and biased. Inconsistent age stratification of enrolled patients and normal populations can also affect SMR results. Young people were associated with less underlying diseases than the elderly. The proportion of young and elderly patients in the enrolled patient population could significantly affect the results of non-tumor causes of death. A study found that congestive heart failure, chronic kidney diseases, peripheral vascular diseases, COPD, diabetes and cerebrovascular diseases were associated with the decreased overall survival of localized kidney cancer patients [27]. In the USA, approximately 2/3 of medicare beneficiaries over age 65 have two or more chronic conditions, with 1/3 having four or more [28]. The increasing multimorbidity trend with age was associated with higher risks of death from non-cancer diseases [29].

Our study firstly provided the most comprehensive description of the risk of death from all causes in patients with different pathological type kidney cancer. Patients were analyzed, respectively, according to their different tumor stages and survival time. However, there were some limitations in our study. Firstly, we used Summary Stage 2000 (1998 +) to distinguish localized and regional cancer, but these classifications may not be the same in different periods. Therefore, the results of this study cannot fully represent the current staging results. Secondly, the treatment information of patients in this database was incomplete. The database only provided radiotherapy and chemotherapy records, but not detailed treatment information. There was no information about immunotherapy or targeted therapy. These treatments might have important impacts on the survival and prognosis of patients. Our results might be influenced by the missing information.

## Conclusion

Our study provides a detailed analysis of the causes of death for patients with localized and regional kidney cancer after diagnosis, and the mortal risk for each cause. Diseases including lung and bronchus cancer, bladder cancer, pancreas cancer, colon and rectum cancer, diseases of heart, COPD, cerebrovascular diseases are the leading causes of death. After identifying the underlying causes of mortality, our study has the potential to facilitate interdisciplinary and multiprofessional collaborations that could significantly enhance prognosis and survival rates. Further research is required to elucidate the mechanisms behind the development of secondary malignancies and non-neoplastic diseases in patients with kidney cancer, as well as to explore differences between clear cell and non-clear cell renal carcinoma.

## Supplementary Information

**Additional file 1.** Raw data used in the study.

## Data Availability

The data of this study are available in the SEER database (https://seer.cancer.gov/). The raw data have been uploaded as the Additional file 1.
